# Attention-dependent coupling with forebrain and brainstem neuromodulatory nuclei differs across the lifespan

**DOI:** 10.1007/s11357-025-01582-0

**Published:** 2025-03-04

**Authors:** Nicholas G. Cicero, Elizabeth Riley, Khena M. Swallow, Eve De Rosa, Adam Anderson

**Affiliations:** Department of Psychology, Cornell University, Ithaca, NY 14853 USA

**Keywords:** Basal forebrain, Locus coeruleus, Functional connectivity

## Abstract

Attentional states reflect the changing behavioral relevance of stimuli in one’s environment, having important consequences for learning and memory. Supporting well-established cortical contributions, attentional states are hypothesized to originate from subcortical neuromodulatory nuclei, such as the basal forebrain (BF) and locus coeruleus (LC), which are among the first to change with aging. Here, we characterized the interplay between BF and LC neuromodulatory nuclei and their relation to two common afferent cortical targets important for attention and memory, the posterior cingulate cortex and hippocampus, across the adult lifespan. Using an auditory target discrimination task during functional MRI, we examined the influence of attentional and behavioral salience on task-dependent functional connectivity in younger (19–45 years) and older adults (66–86 years). In younger adults, BF functional connectivity was largely driven by target processing, while LC connectivity was associated with distractor processing. These patterns are reversed in older adults. This age-dependent connectivity pattern generalized to the nucleus basalis of Meynert and medial septal subnuclei. Preliminary data from middle-aged adults indicates a transitional stage in BF and LC functional connectivity. Overall, these results reveal distinct roles of subcortical neuromodulatory systems in attentional salience related to behavioral relevance and their potential reversed roles with aging, consistent with managing increased salience of behaviorally irrelevant distraction in older adults. Such prominent differences in functional coupling across the lifespan from these subcortical neuromodulatory nuclei suggests they may be drivers of widespread cortical changes in neurocognitive aging, and middle age as an opportune time for intervention.

**Supplementary Information:**

The online version contains supplementary material available at 10.1007/s11357-025-01582-0.

## Introduction

To produce adaptive behavior, the brain balances the prioritization of goal-relevant information with the need to respond to changing environmental demands [1]. Depending on the situation, systems that process task-relevant information may predominate or give way to systems that promote shifts in cognitive states [2, 3]. For example, when driving towards a traffic light, a yellow light could signal a switch from maintaining a steady speed to assessing if you need to slow down. The flexibility to dynamically shift attentional states is vital for cognitive functioning [4] and is accompanied by prominent changes across the adult life span [5–7]. While behavioral relevance involves neocortical systems to regulate attention and action [8–12], subcortical modulatory systems tune the inter and intra-region dynamics for attention. It is in these regions where there are the first signs of aging-related pathology [13–17].

Subcortical neuromodulatory systems are the foundation of short-term shifts in the flexible tuning of brain networks towards objects of attention [18, 19]. Influencing distributed processes throughout the brain, the basal forebrain (BF) and locus coeruleus (LC) release neuromodulators, adjusting the responsiveness of target region neurons. The LC releases norepinephrine (NE) [18, 19] and the BF, which consists of four regions (Ch1-3 = medial septum (MS); Ch4 = nucleus basalis of Meynert (nbM)), releases acetylcholine (ACh) and gamma-aminobutyric acid (GABA) [20, 21]. These systems work together, with BF-GABA proposed to initiate a rapid response to salient stimuli, which then activates the BF-ACh and LC-NE systems to sustain attentional, sensory, and information processing when needed [22]. Critically, both the LC and BF have particularly prominent innervations in the hippocampus (HPC) and posterior cingulate cortex (PCC), which are involved in spatiotemporal representations of memory and shifts to and from internally driven cognition, respectively [8–12, 23]. The HPC and PCC have also shown significant functional connectivity with these neuromodulatory regions [12, 23, 24] and are subject to prominent age-related changes [25–28]. The release of NE and ACh thus contributes to the PCC and HPC’s respective roles in cognition and may play an important role in understanding neurocognitive aging [5–7, 29].

The LC and BF nuclei not only project to the HPC and PCC but are themselves strongly interconnected. The LC is the primary source of noradrenergic innervation to the nbM [11] and shows significant functional connectivity [24]. NE and ACh have complementary roles in computing uncertainty in the environment, such that ACh signals expected uncertainty, suppressing expectation-driven information in environments with predictability. In contrast, NE signals unexpected uncertainty in which sensory information violates top-down expectations [30]. Additionally, NE flattens low-dimensional energy landscapes of cortical dynamics, reducing the difficulty of switching brain states, whereas ACh has the opposite effect by deepening these landscapes [31]. Overall, the LC and BF are thought to be synergistically connected and contribute to flexibility in the allocation of attention related to moment-to-moment changes in behavioral salience. Despite this, it remains unclear how this interconnected network of neuromodulatory nuclei and their cortical afferents together contribute to moment-to-moment attentional processing.

Assessing these neuromodulatory systems in a context where they naturally change provides an opportunity to better understand their functional dynamics. One such context is healthy aging, in which there are changes to attentional orienting [32], distractibility [33], and to the structural integrity of these neuromodulatory nuclei [34]. The LC and BF are some of the first regions to show evidence of pathology in aging [13–17] and this likely creates cognitive vulnerabilities years prior to the development of cognitive impairment, as has been demonstrated in Alzheimer’s Disease [34–37]. Despite evidence of structural alterations in these neuromodulatory nuclei, it is unknown how these nuclei function within a network and how network structure differs with age particularly in a context that requires adjusting functional dynamics to changes in attentional salience.

One of the challenges of examining the contributions of these nuclei is their small size and location in regions that were traditionally challenging to characterize functionally. Advances in magnetic resonance imaging (MRI) allow us to reliably investigate these nuclei in humans and their age-related modification. Turbo-spin echo (TSE) imaging provides a technique for structural localization of the small LC region [24, 38] and multi-echo fMRI has been demonstrated to obtain high signal in the BF and LC [12, 24]. We use these tools to assess the age-related differences in the interplay of the LC and BF nuclei and their regulation of their common cortical afferents, the HPC and PCC, during a task that dynamically modifies attentional and behavioral relevance. Given that resting-state functional connectivity studies have found LC-nbM connectivity [11, 24], the LC and nbM regulate attentional processing, and the LC and nbM’s cortical afferents are involved in memory and internally directed attention [12, 24, 39], we hypothesize that these subcortical neuromodulatory nuclei will have significant attention-dependent functional connectivity with their cortical afferents, as well as between subcortical nuclei.

A new understanding of age-related differences in these neuromodulatory systems that are the first signs of pathological aging is important for testing and understanding the efficacy of clinical interventions. Neuromodulation techniques may be efficacious by targeting brain networks rather than just the focal region receiving stimulation [40–42]. By assessing functional connectivity patterns through seeding of both subcortical neuromodulatory (sources) and their cortical (afferent targets) regions, we examine brain networks that may be regulated by, and in turn may regulate, subcortical neuromodulatory influences. Revealing the potential complexity of perturbations to neuromodulatory system dynamics affords a deeper understanding of potential contributions of each node in subcortical-cortical networks, and their age-associated differences, which is a critical step to targeting during normal and pathological age-related cognitive decline.

## Methods

### Participants

We examined 85 participants (36 younger adults, 14 middle-aged adults, 35 older adults) who completed this task as a part of a larger study that included neuropsychological assessment, structural and functional MRI scans, and several other cognitive tasks. All participants provided written informed consent, and all study procedures were approved by the Cornell University Institutional Review Board. Middle-aged adults were studied, but due to the small sample size, data from these participants are only included in supplemental analyses (Supplemental Figs 1-3). Pupillary data and analyses from the same participants have been previously published in Riley et al. [43, 44]. MRI data was not available for 18 participants because they did not complete all of the task or the acquired data was unusable due to technical problems. Of the subjects that were included in the final dataset, we checked the average framewise displacement across subjects to assess for any differences in data quality. There were no statistically significant differences in subject motion during runs of the target detection task across age groups (young: mean = 0.065 mm, std = 0.01mm; middle: mean = 0.078 mm, std = 0.01 mm; old: mean = 0.10 mm, std = 0.02 mm), indicating no systematic differences in data quality amongst subjects whose data was included in the final sample.

### Participant characteristics

Our ultimate dataset consisted of 30 younger (aged 19–45; average 25.16 years old; 64.5% female), 14 middle-aged adults (aged 46–65; average 58.14 years old; 61.2% female), and 23 older adults (aged 66–86; average 70.48 years old; 50% female) for a final sample of 67 healthy adults. Age bands were chosen based on US census guidelines. Participants were screened for pregnancy, diagnosed cognitive impairment, neurological disease, street drug use, psychiatric medication, head injury, ocular disease, and had vision and hearing that were normal or correctible-to-normal. Left-handed participants made up 6% of younger adults (two participants), 14% of middle-aged adults (two participants), and 8% of older adults (two participants). Younger, middle-aged, and older adults had an average of 17.2 years (SD = 3.1), 17.2 years (SD = 3.1), and 17.5 years (SD = 2.9) of education, respectively. All participants were screened for cognitive impairment with the Montreal Cognitive Assessment. Younger adults had an average score of 27.9 (SD = 1.6, range 25–30, zero below cutoff), middle-aged adults had an average score of 27.2 (SD = 1.9, range 25–30, one below cutoff), and older adults had an average score of 27.0 (SD = 2.4, range 25–30, four below the cutoff after adjustment for years of education). None had a diagnosis of cognitive impairment of any kind. Participants were also given the Trail Making Test Part B, with an average time of 70.3s (SD = 23.7, range 41–119) for younger adults, 81.7s (SD = 37.8, range 39-177) for middle-aged adults and 95.4s (SD = 24.0, range 50–151) for older adults. While older adults had slower times to completion as expected, none were longer than the predefined cutoff of 180 seconds.

Any participants that used vision correction were either given MR-safe lenses during testing, or, if they only used vision correction for reading, were given a brief vision test before entering the scanner to ensure that they would be able to see the task stimuli in focus.

### Task overview

Detailed descriptions of the task and stimuli are presented in Riley et al. [43, 44] but are recounted here. Participants were instructed to remember a series of pictures for a later memory test while performing a target discrimination task. During the target discrimination task, participants were instructed to listen for two types of tones (low and high) and press a button when they heard the target, but not the distractor, tone. Unlike a traditional go/no-go task, target and distractor trials were equiprobable. During each of four runs, the participants were told which tone (low or high, order alternating and counterbalanced) was the designated target tone for that run. As such, the behavioral relevance and salience of tones was systematically manipulated across the task duration. Detecting a target in the task has previously been shown to increase the attentional and memory salience for concurrently presented events, elicit activity in the LC relative to both distractor and no tone conditions [45–47], elicit increases in phasic pupillary responses [44, 48], and enhance functional connectivity between the ventral visual cortex and hippocampus [49].

### Task stimuli

Tone stimuli were either high (1200 Hz) or low (400 Hz) and had a duration of 60 ms. To maintain a consistent level of luminance and cognitive engagement across the target detection task, background visual stimuli were displayed. Visual stimuli consisted of 144 color pictures and were evenly divided among pictures of faces, objects, and scenes. One hundred and forty-four additional scrambled image masks derived from the source images were generated. The images were acquired from online resources [50] (http://vision.stanford.edu/projects/sceneclassification/resources.html) and personal collections. After each trial, the scrambled masks were presented to maintain luminance. Scrambled images were created by dividing an image into 256 squares and randomly shuffling them. Pixel intensities, both mean and variance, were matched across images using the SHINE toolbox [51].

### Task procedure

The target discrimination task was completed as part of a longer MRI protocol. Each run lasted 6 min 47 s, for a total duration of less than 30 min across all four runs. Before the experiment, participants practiced the task. Task trials were designated as target, distractor, or no tone trials in equal numbers and the target tone alternated across blocks. The starting target tone was counterbalanced across participants. Prior to the task, participants were instructed that memory for the pictures would be tested after the scan session. Participants were asked to maintain fixation on a dot (0.25 visual degree diameter, red) at the center of the picture throughout the task. Each trial lasted 1.25 s long, with one image (7 × 7 visual degrees; 256 × 256 pixels) presented for 625 ms immediately followed by a scrambled version of that same image for another 625 ms, and in some cases further scrambled images, also for 625 ms per scrambled image. The rapid timing of these images and scrambled images encouraged vigilance and rapid response times.

Each run consisted of 144 trials. All 144 images were presented one time per block for a total of four repetitions across blocks and 576 total task trials. Each trial is either a high- or low-pitch or no tone played. Participants were instructed to press a button with their dominant hand pointer finger when they heard the designated target tone for that run and to make no motor response on trials with a distractor tone or no tone. The target, distractor, and no-tone trials were equiprobable, with a behavioral response required on target trials and withheld on distractor and no tone trials. Each run consisted of 164 scrambled images without sound, with at least one scrambled image presented between each trial to increase the unpredictability of the task trials. The median inter-trial interval of non-scrambled images was 2.5 s. Tone volume was adjusted during a mock scan to ensure that participants were able to hear both tones over scanner noise.

The timing and order of the trials was optimized using the AFNI function make_random_timing to maximize orthogonality of overlapping BOLD responses across trials and minimize the amount of unexplained variance in a simulated task. Inter-trial intervals were filled with scrambled images, as described above.

### MRI acquisition and preprocessing

Imaging was carried out at the Cornell University MRI facility with a GE discovery MR750 3T scanner and a 32-channel head coil. Participants laid supine on the scanner bed with their head supported and stabilized. Ear plugs, headphones, and a microphone were used to reduce scanner noise, allow the participant to communicate with the experimenters, and to present auditory stimuli during the tasks. Visual stimuli were presented with a 32” Nordic Neuro Lab liquid crystal display (1920 × 1080 pixels, 60 Hz, 6.5 ms g to g) located at the back of the scanner bore and viewed through a mirror attached to the head coil. Pulse rate and respiration were recorded throughout all scans.

The imaging protocol consisted of a multi-echo acquisition (TR = 2500; TEs = 12.3, 26.0, and 40.0 ms; flip angle = 90°; matrix = 72 × 72; fov = 21 cm; 3.0 mm isotropic voxels; 44 slices; 159 volumes per run) and a structural T1-weighted MPRAGE (TR/TE = 7/3.42 ms; flip angle = 7°; matrix = 256 × 256; fov = 24 cm; 1 mm isotropic voxels). High-resolution images of the LC were acquired with neuromelanin-sensitive T1-weighted turbo-spin echo (TSE) structural scans (scan resolution = 512 × 512 mm; 13 slices; 3.0 mm × 0.43 mm × 0.43 mm; fov = 22.0 mm × 132.00 mm; TE = 11.26 ms; TR = 700 ms; flip angle = 120°). Visual stimuli were presented on a screen with a mirror, auditory stimuli were presented to participants via headphones, and participants responded to various tasks in the scanner by pressing a button box. Participants subsequently completed additional anatomical and functional scans that will be reported separately.

Preprocessing of the structural MRI images included normalization, skull stripping, and segmentation. Processing of the multi-echo EPI data was performed using a standard preprocessing pipeline from AFNI (afni_procy.py; tedana.py, Version 2.4 beta 11) that consisted of the following steps. First, spikes in each voxel’s time series from the EPI data were truncated using 3dDespike. Second, slice-time correction was performed using 3dTshift. Third, the EPI data was aligned to the MPRAGE using align_epi_anat.py. Fourth, the MPRAGE was warped to MNI N27 space. Then, all EPI transformations were concatenated and the EPI data was warped to MNI space in a single step. A brain mask of the EPI data was then created before a multi-echo combination. The multi-echo combination was completed using AFNI’s multi-echo ICA script (tedana.py, Version 2.4 beta 11). Finally, each voxel’s time series was scaled to have a mean of 100 (arbitrary units). Altogether, the afni_proc.py blocks implemented were in the following order: despike, tshift, align, tlrc, volreg, mask, combine, and scale. After preprocessing, the EPI data was spatially smoothed (FWHM = 6 mm). This minimal amount of smoothing has been shown to not critically change the ability to isolate LC activity [24]. Quality control checks were completed at each stage in preprocessing to ensure accurate completion of each step.

### MRI processing: regions of interest

**Fig. 1:**
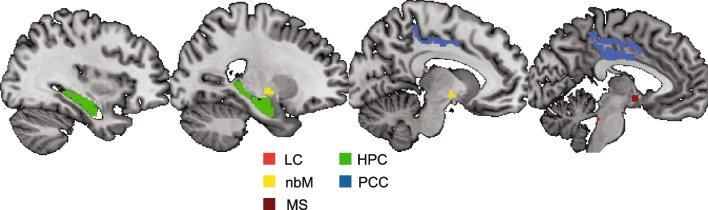
All ROIs in standard MNI space. HPC and PCC ROIs are segmented from FreeSurfer’s parcellation. nbM and MS ROIs are from a probabilistic atlas. LC ROIs are derived from neuromelanin-sensitive TSE scans. The HPC, PCC, nbM, MS, and LC ROIs are from an example subject in standard MNI space. LC, locus coeruleus; HPC, hippocampus; nbM, nucleus basalis of Meynert; PCC, posterior cingulate cortex; MS, medial septum

LC ROIs were created for each participant from their putative neuromelanin sensitive TSE scan as described in Turker et al. [24]. Briefly, all non-brainstem and non-4th ventricle voxels were masked out of the TSE scans. Each slice was then mean centered by subtracting the mean signal intensity of voxels within the brainstem and then the LC was manually defined by two independent rates (NGC and ER) based on criteria in Turker et al. [24]. Consistency across the two independent raters was checked and then the intersection of the manually defined LC ROIs from the two independent rates was used for the final LC ROI for all subsequent analyses. The ROIs for the BF were taken from a probabilistic ROI obtained from a previous study [52]. Separate BFs for BF Ch1-3 (MS) and BF Ch4 (nbM) were obtained. Both BF ROIs were warped to native N27 space and thresholded. Left and right hippocampal and left and right posterior cingulate cortical (PCC) ROIs for each participant were obtained from FreeSurfer’s automatic parcellation. Volumes were reviewed for accuracy and masks were manually edited if necessary. After extraction, each participant’s HPC and PCC ROIs were thresholded to eliminate occasional nonzero voxels introduced during the warping process. Probabilistic HPC and PCC ROIs were obtained by finding the union set of all participants’ specific ROIs. ROIs in standard MNI space are shown in Fig. 1. As HPC volume has shown to change with age [27], we ensured good overlap in HPC masks between each age group. The average HPC mask for each age group was formed and then the dice coefficient between all pairs of age groups was calculated to assess overlap between groups. All age group pairs showed good overlap in average HPC masks (young-middle: dice = 0.751; young-old: dice = 0.734; middle-old: dice = 0.729).

### MRI processing: task-dependent functional connectivity

To assess task-dependent functional connectivity between the LC, BF, and other target ROIs, we conducted a generalized psychophysiological interaction (gPPI) analysis. This analysis was used because of the three task conditions and rapid event-related design of our target detection task [53–58]. GPPI allowed us to characterize changes in LC, nbM, and MS functional coupling with other areas as they relate to the three-target detection task trial conditions within subjects (1: target, 2: distractor, 3: no tone), whereas the bivariate, correlation-based analysis of functional connectivity is unconstrained by task demands and thus insufficient to reveal the influence of task demands on functional connectivity.

**Fig. 2:**
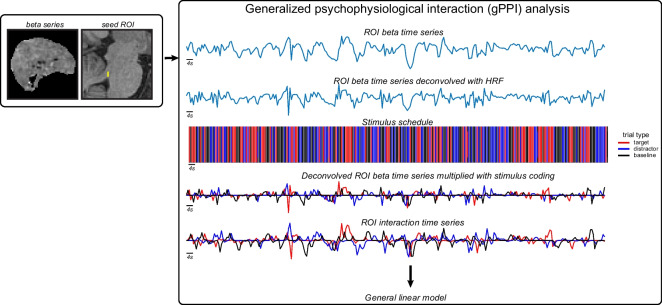
Exemplar inputs for generalized psychophysiological interaction analysis. (*left*) The voxelwise beta series is computed using a least-squares-sum estimation approach and for a given seed ROI (shown in the figure is the LC). The beta series is extracted from each participant’s ROI. (*right*) After the ROI beta time series is extracted, a stimulus-specific canonical HRF is deconvolved with the ROI beta time series. For a given stimulus schedule, the deconvolved ROI beta time series is multiplied for the stimulus schedule for each trial type (target [red], distractor [blue], baseline [black]), resulting in three stimulus-specific deconvolved ROI beta time series. Each time series is then convolved with a canonical HRF, resulting in an ROI interaction time series for each trial type that are used as inputs for a whole-brain GLM analysis

For all seed ROIs, the seed region ROI BOLD was extracted in MNI space (Fig. 1) to keep the spatial transformation consistent between the seed ROI and the whole-brain functional data that the gPPI is performed over. The steps for the gPPI analysis are as follows (Fig. 2). Briefly, the voxelwise beta series was computed using a least-squares sum (LSS) estimation approach (3dLSS in AFNI), generating one beta per trial for each run. Next, the beta series from each participant’s LC ROI was extracted (3dmaskave in AFNI). A canonical gamma hemodynamic response function (HRF) was convolved with the participant’s target tone stimulus timing file as input. Then, the LC beta series was deconvolved with the stimulus-specific gamma HRF (waver in AFNI). A stimulus coding file was then generated that was the length of the number of trials in a single run, but each time point was 1 if that trial was a target tone or 0 if that trial was a distractor tone or no tone. The interaction between the deconvolved LC beta series and the stimulus coding file was then assessed by multiplying the two files (1deval -expr “a*b” in AFNI). The interaction time series was then obtained by convolving the previous step’s output with a canonical gamma HRF.

The above steps were repeated for each participant’s distractor and baseline trial time series separately, resulting in each participant having three separate interaction beta time series for each of the three task conditions. These interaction time series were then entered into a general linear model with the LC beta series (3dDeconvolve in AFNI), and interaction term beta weights were extracted for each trial type per subject. Interaction term beta weights (referred to as “gPPI parameter estimates” going forward) for each trial type are interpreted as that specific trial’s task-dependent functional coupling for all voxels with the LC for that subject. The above steps were repeated with the nbM and MS ROIs as seeds to generate attention-dependent functional connectivity estimates across all three subcortical and brainstem neuromodulatory regions within each participant.

We operationally define target-related connectivity as task-dependent functional connectivity (gPPI parameter estimates) that is greater during target relative to distractor trials, and we define distractor-related connectivity as connectivity that is greater during distractor relative to target trials. These definitions are useful for quantifying how trial-by-trial connectivity between neuromodulatory regions and its afferents support these two different aspects of the target detection task. Additionally, gPPI provides information about the relative change in functional connectivity between task conditions, but not absolute functional connectivity in a single task condition [53–55]. From this analysis, we hope to elucidate not the absolute functional connectivity during different task conditions and in different age groups, but rather the relative difference in connectivity between task conditions and how this relative difference changes across age groups.

### Statistical analysis: models

Several linear mixed effects models were completed in AFNI (3dLME) to assess gPPI parameter estimates in relation to several within- and between-subjects variables, while taking into account the random effect of subject and sex. A linear mixed effects model was completed on the gPPI parameter estimates to assess differences in task-dependent functional coupling for each seed ROI in relation to trial type condition (target, distractor, no tone) and age group (young, old), with random effects of subject and sex. Each linear mixed effects model for each seed ROI yielded whole-brain voxelwise results for the main effects of trial type and age group, as well as the interaction between trial type and age group. Cluster correction on the linear mixed effects results was completed to find the cluster size threshold (in voxels) and to find significant clusters (3dClusterize AFNI). Small-volume correction was completed using AFNI’s 3dClustSim to compute a threshold for a voxelwise *p*-value given the surviving cluster size. Because prior reports on these data reported activation for several of the regions of interest (for LC and nbM main effects see: Riley et al., [43]), we focus analyses on connectivity.

For each seed ROI, a significant task-dependent functional connectivity was assessed by checking for clusters within the ROIs (Fig. 1) that remained after cluster correction and small volume correction. Note that significant clusters do not encompass the entire region of interest but rather contain only the group of voxels with significant task-dependent functional connectivity. This allows for an assessment of different connectivity patterns between subareas within each ROI. To assess differences in task-dependent functional connectivity from significant clusters across continuous age, we extracted gPPI parameter estimates per subject and assessed the relationship between gPPI parameter estimates and years of age post hoc. We computed robust linear regression between gPPI parameter estimates and years of age to reduce the impact of outliers. Significance tests for these correlations are performed to test the null hypothesis that the distributions underlying the samples are uncorrelated and normally distributed. Correction for multiple comparisons for between age group analyses was completed using Bonferroni correction to correct for the number of *t*-tests performed and the number of linear mixed effects models computed across the three seed ROIs. Additional exploratory gPPI analyses included a smaller reference sample of middle-aged adults are provided in Supplementary Materials.

## Results

### Target detection task behavioral performance

The full analysis of behavioral performance on the target detection task is provided in detail in two other publications [43, 44] but reviewed here for reference. By design, due to the simple nature of the task, we observed no significant age differences in accuracy on the task [43, 44]. As was expected due to psychomotor slowing, despite equivalent accuracy, older adults exhibited a significantly slower reaction time on accurate trials than younger adults [44]. Consistent with the low demands of the task, we observed a largely similar task performance across younger and older adults, and thus connectivity differences across age groups were not a consequence of differential performance.

### LC-seeded network

To determine how LC functional connectivity with its known afferents differed across conditions and age groups, a linear mixed effects model was used. GPPI interaction parameter estimates for each trial, by tone type and age group, and including their interaction (tone × age group) were entered into the model.

#### LC-BF functional connectivity

Significant clusters in the nucleus basalis of Meynert (nbM) represented significant LC-nbM functional connectivity changes. There was no significant cluster for the main effect of age group, nor the main effect of trial type. There was a significant nbM cluster for the age-by-trial type interaction (*X*, *Y*, *Z* = −22, 6, −7; cluster size = 7 voxels; *z* = −2.83; *p* = 0.038), indicating a difference in task-dependent LC-nbM coupling across age groups.

**Fig. 3:**
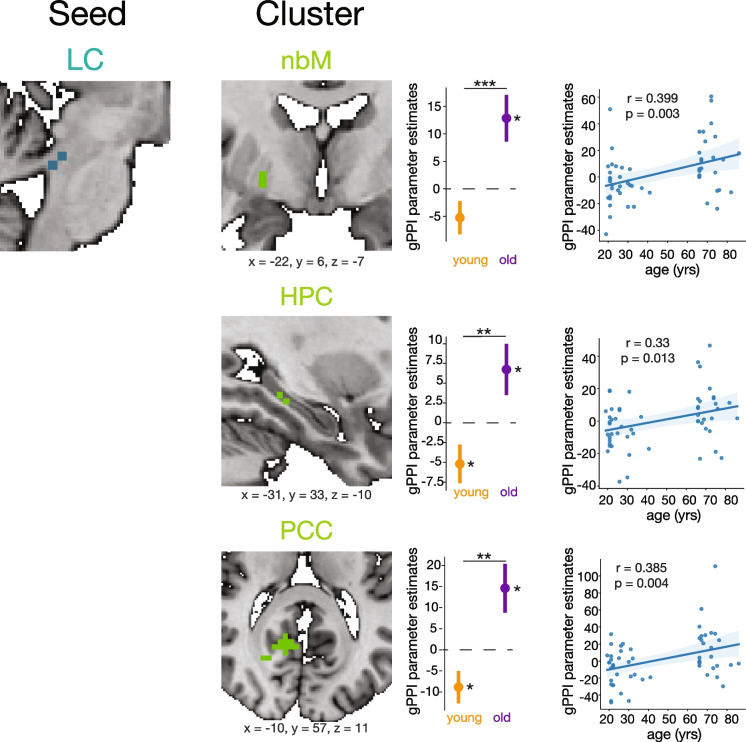
LC-seeded task-dependent functional connectivity across the lifespan. Regions of interest with a significant age-by-trial type (target vs. distractor) interaction cluster for task-dependent functional connectivity with the LC are shown. Greater gPPI estimates indicate greater functional connectivity to targets than distractors. Clusters are displayed and coordinates are listed in MNI N27 space. Each cluster represents the psychophysiological interaction parameter estimates of the difference between target and distractor trials. An asterisk to the right of a single-colored bar indicates a significant difference from zero (no task-dependent connectivity changes) with a one-sample *t*-test. Asterisks spanning two colored bars indicate a significant difference across two age groups computed with a two-sample *t*-test. Correlations between gPPI parameter estimates and age are also shown with the corresponding Pearson coefficient and *p*-value. There was no significant LC-MS functional connectivity. LC, locus coeruleus; HPC, hippocampus; nbM, nucleus basalis of Meynert; PCC, posterior cingulate cortex

To investigate the age-by-trial type interaction further, we extracted gPPI parameters from all subjects within the significant nbM cluster. We first computed one-sample *t*-tests within each age group to assess if task-dependent coupling within a given age group was stronger in targets or distractors. We found that in younger adults the LC-nbM connectivity was marginally greater during distractors (*t* = −1.706, *p* = 0.098) and in older adults was significantly greater during targets (*t* = 2.967, *p* = 0.007) (Fig. 3). In comparing across age groups, LC-nbM coupling in older adults was significantly greater in targets versus distractors compared to younger adults (*t* = −3.48, *p* < 0.001). The change in LC-nbM functional connectivity between targets and distractors had a significant correlation with age (*r* = 0.399, *p* = 0.003). Task-dependent LC-nbM coupling trended towards being greater during distractor trials in younger but switched to being greater during target trials in older adults.

There was no significant LC-MS (BF Ch1-3) coupling for any main effects nor interactions. This aligns with previous literature indicating structural connections between the LC and nbM [11], but not between the LC and MS.

#### LC-hippocampus (HPC) functional connectivity

Significant clusters in the HPC represent significant LC-HPC functional connectivity differences across age group or trial type. There was a significant cluster in the HPC for the main effect of age (*X*, *Y*, *Z* = −16, 30, −7; cluster size = 61 voxels; F = 15.01; *p* = 0.008). There was no significant cluster in the HPC for the main effect of trial type. However, there was a significant HPC cluster for the trial type-by-age interaction (*X*, Y, *Z* = −31, 33, −10; cluster size = 6 voxels; *z* = -2.67; *p* = 0.04).

Upon further investigation, LC-HPC coupling in younger adults indicated connectivity significantly greater during distractor relative to target trials (*t* = −2.098, *p* = 0.044). In older adults, LC-HPC coupling was significantly greater during target relative to distractor trials (*t* = 2.036, *p* = 0.052). LC-HPC coupling for targets compared to distractors was significantly greater in older adults compared to younger (*t* = −2.94, *p* = 0.0048). (Fig. 3). The change in LC-HPC functional connectivity between targets and distractors had a significant correlation with age (*r* = 0.33, *p* = 0.013). Similar to LC-nbM connectivity, task-dependent LC-HPC coupling was greater during distractor trials in young adults, but in older adults was greater during target trials.

#### LC-posterior cingulate cortex (PCC) functional connectivity

Significant clusters in the PCC represent significant LC-PCC functional connectivity changes. There was a significant cluster in the PCC for the main effect of trial type (*X*, Y, *Z* = 14, 51, 8; cluster size = 10 voxels, *F* = 4.72; *p* = 0.049). There was no significant cluster in the PCC for the main effect of age group. There was a significant PCC cluster for the trial type-by-age interaction (*X*, Y, *Z* = −10, 57, 11; cluster size = 39 voxels; *z* = −2.16; *p* = 0.03).

One-sample *t*-tests revealed that, in younger adults, LC-PCC coupling was greater during distractor trials (*t* = -2.276, *p* = 0.03), whereas LC-PCC coupling in older adults was greater during target trials (*t* = 2.481, *p* = 0.02). LC-PCC coupling for targets compared to distractors was significantly greater in older adults compared to younger adults (*t* = −3.422, *p* = 0.001) (Fig. 3). The change in LC-PCC functional connectivity between targets and distractors had a significant positive linear correlation with age (*r* = 0.385, *p* = 0.004). The results overall indicate that LC-PCC coupling was greater during distractor trials in younger adults but in older adults was greater during target trials.

### nbM-seeded network

To determine how nbM functional connectivity with its known afferents differed across conditions and age groups in our orienting task, a linear mixed effects model was used. GPPI parameter estimates for each trial, by tone type and age group, and including their interaction (tone × age group) were entered into the model.

#### nbM-LC functional connectivity

Significant clusters in the LC represent significant nbM-LC functional connectivity changes. There were no significant LC clusters for the main effect of age group nor trial type, but there was a significant LC cluster for the trial type-by-age interaction (*X*, *Y*, *Z* = −6, 39, −21; cluster size = 33 voxels; *z* = 3.306; *p* = 0.028).

**Fig. 4:**
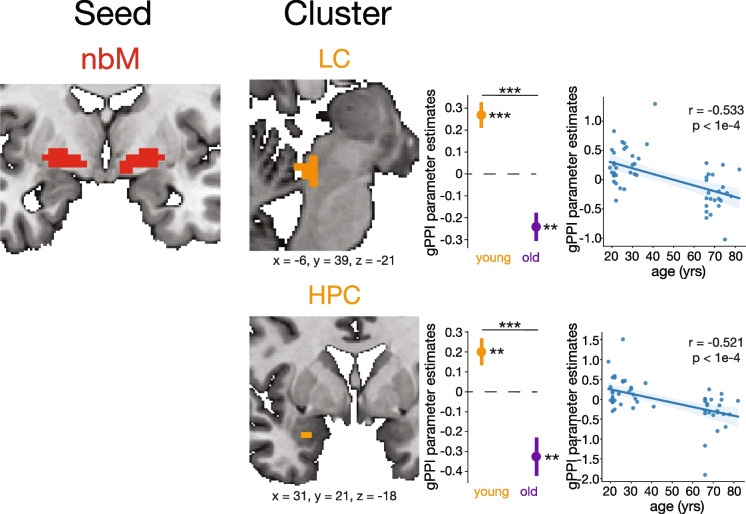
nbM-seeded task-dependent functional connectivity across the lifespan. Regions of interest with a significant age-by-trial type (target vs. distractor) interaction cluster for task-dependent functional connectivity with the nbM are shown. Greater gPPI estimates indicate greater functional connectivity to targets than distractors. Clusters are displayed and coordinates are listed in MNI N27 space. Each cluster represents the psychophysiological interaction parameter estimates of the difference between target and distractor trials. An asterisk to the right of a single-colored bar indicates a significant difference from zero (no task-dependent connectivity changes) with a one-sample *t*-test. Asterisks spanning two colored bars indicate a significant difference across two age groups computed with a two-sample *t*-test. Correlations between gPPI parameter estimates and age are also shown with the corresponding Pearson coefficient and *p*-value. There was no significant nbM-MS or nbM-PCC functional connectivity. LC, locus coeruleus; HPC, hippocampus; nbM, nucleus basalis of Meynert

One-sample *t*-tests revealed that in younger adults, nbM-LC connectivity was significantly greater during target relative to distractor trials (*t* = 4.536, *p* < 1e-4) (Fig. 4). In older adults, nbM-LC coupling reversed and was instead stronger during distractor trials (*t* = −3.664, *p* = 0.0013). nbM-LC coupling between targets and distractors was greater in younger adults compared to older adults (*t* = 5.721, *p* < 1e-6). The change in nbM-LC functional connectivity between targets and distractors resulted in a significant negative linear correlation with age (*r* = −0.553, *p* < 1e-4). Task-dependent nbM-LC coupling, with nbM as the seed, was greater during target trials in young adults but strongly reversed in old age to be greater during distractor trials.

## *nbM-HPC* functional connectivity

Significant clusters in the HPC represented significant nbM-HPC functional connectivity changes. There was a significant HPC cluster for the main effect of trial type group across all age groups (*X*, *Y*, Z = −25, 6, −37; cluster size = 19 voxels; *F* = 5.219; *p* = 0.0487) that was marginally significant after multiple comparisons correction. Additionally, there was a HPC cluster for the main effect of age group (*X*, *Y*, *Z* = −22, 6, −22; cluster size = 10 voxels; *F* = 7.048; *p* = 0.0414) that was marginally significant after multiple comparisons correction. Finally, there was a significant HPC cluster for the trial type-by-age interaction (*X*, *Y*, *Z* = 31, 21, −18; cluster size = 70 voxels; *z* = 3.365; *p* = 0.022).

Upon further investigation, nbM-HPC coupling was significantly greater during target trials for young adults (*t* = 2.949, *p* = 0.006). By contrast nbM-HPC coupling was significantly greater during distractor trials for older adults (*t* = -3.335, *p* = 0.003) (Fig. 4). Across age groups, nbM-HPC coupling between targets and distractors in older adults significantly decreased compared to younger adults (*t* = 4.563, *p* < 1.0e-4). The change in nbM-HPC functional connectivity between targets and distractors had a significant negative correlation with years of age (*r* = −0.52, *p* < 1.0e-4). Task-dependent nbM-HPC coupling was greater during target trials in younger adults but markedly reversed to be greater during distractor trials in older adults.

There was no significant nbM-MS task-dependent coupling for any main effects nor interactions. This aligns with the lack of previous literature indicating structural connections between the nbM and MS [12, 15]. There was also no significant nbM-PCC coupling for any main effects nor interactions.

### MS-seeded network

GPPI interaction parameter estimates for trials, by tone type and age group, and their interaction (tone × age group) during the target detection task were entered into a linear mixed effects model to assess MS task-dependent functional connectivity with known afferents.

## *MS-HPC* functional connectivity

Significant clusters in the HPC represented MS-HPC functional connectivity changes across task conditions. There was a significant HPC cluster for the main effect of trial type (*X*, *Y*, *Z* = −16, 3, 18; cluster size = 11 voxels; *F* = 5.87, *p* = 0.049). There was also a significant HPC cluster for the main effect of age group (*X*, *Y*, *Z* = −31, 6, −28; cluster size = 40 voxels; *F* = 12.848, *p* = 0.015). There was a significant HPC cluster for the age-by-trial type interaction (*X*, *Y*, *Z* = −40, 21, −15; cluster size = 63 voxels; *z* = 2.707; *p* = 0.028).

**Fig. 5:**
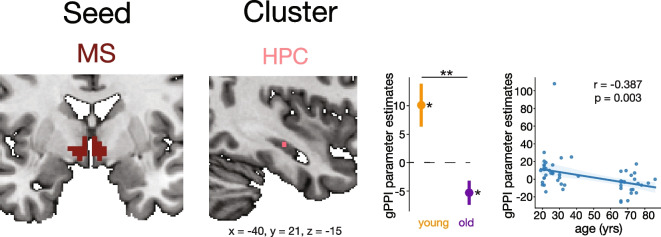
MS-seeded task-dependent functional connectivity across the lifespan. Regions of interest with a significant age-by-trial type (target vs distractor) interaction cluster for task-dependent functional connectivity with the MS are shown. Clusters are displayed and coordinates are listed in MNI N27 space. Greater gPPI estimates indicate greater functional connectivity to targets than distractors. Each cluster represents the psychophysiological interaction parameter estimates of the difference between target and distractor trials. An asterisk to the right of a single-colored bar indicates a significant difference from zero (no task-dependent connectivity changes) with a one-sample *t*-test. Asterisks spanning two colored bars indicate a significant difference across two age groups computed with a two-sample *t*-test. Correlations between gPPI parameter estimates and age are also shown with the corresponding Pearson coefficient and *p*-value. There was no significant MS-LC, MS-nbM, nor MS-PCC functional connectivity. MS, medial septum; HPC, hippocampus

One-sample *t*-tests revealed that in young adults, MS-HPC coupling was greater during targets relative to distractor trials (*t* = 2.651, *p* = 0.0127). In older adults, MS-HPC coupling was instead greater during distractors trials (*t* = −2.467, *p* = 0.0211). MS-HPC coupling differences between targets and distractors in older adults was significantly decreased compared to younger adults (*t* = −3.30, *p* = 0.0017) (Fig. 5). The change in MS-HPC functional connectivity between targets and distractors had a significant negative linear correlation with age (*r* = −0.387, *p* = 0.003).

In line with the known lack of structural connectivity of the MS, there were no significant MS-LC [11], MS-nbM, nor MS-PCC task-dependent coupling for the main effect of age and trial type, nor the interaction of age and trial type.

### Exploration of network changes relative to a reference middle-aged sample

Due to the small middle-aged sample size (*n* = 15) and skewed distribution (aged 46–65; average 58.14 years old), we did not include middle-aged adults in the main analyses. Preliminary analyses exploring the possible lifespan development of these subcortical networks suggest potential nonlinear changes across the lifespan in how these subcortical networks might differ in middle age, but with an overall profile more similar to younger adults (Supplemental Figs 1-3).

### Summary of age-related differences in network configuration

**Fig. 6:**
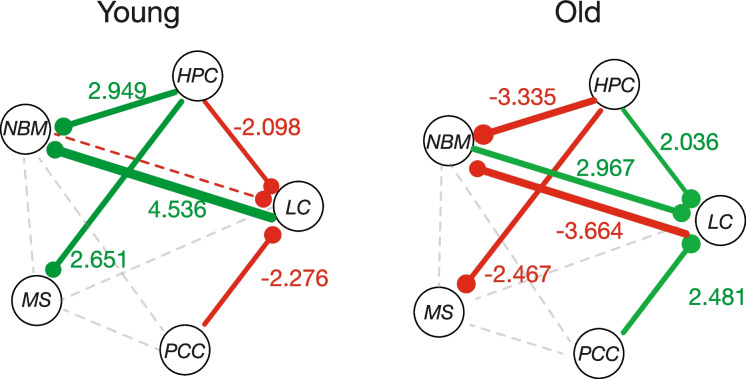
Diagram summarizing age related differences in task-based functional connectivity. Colored lines indicate a significant relationship between functional coupling and task. For colored lines, line thickness and the values along the lines indicate the strength of the relationship, as measured by the *t*-statistic of the task-dependent coupling in each group calculated from a one-sample *t*-test against a mean of zero. Green lines indicate greater coupling during targets compared to distractors. Red lines indicate greater coupling during distractors compared to targets. Grey dotted lines indicate a non-significant influence of task on functional coupling (*p* > 0.1). Color-dotted lines indicate a marginally significant relationship between functional coupling and task that had a *p*-value > 0.05 and *p*-value < 0.1. The seed region is represented by the filled circle at the end of the red or green line. For example, in young adults, nbM-LC connectivity with the nbM as the seed region was greater for targets than distractors. LC, locus coeruleus; nbM, nucleus basalis of Meynert; MS, medial septum; HPC, hippocampus; PCC, posterior cingulate cortex

In younger adults, we observed significant task-related functional coupling in all seed regions, with the majority of task-dependent connections stronger in target (behaviorally salient) trials. This network structure is significantly different in older adults, with a complete reversal in task-related functional coupling in old age (Fig. 6).

## Discussion

We examined how age is associated with altered functional coupling between known afferent projection sites of noradrenergic (LC) and cholinergic (BF) subcortical nuclei in the context of a target discrimination task that manipulated behavioral salience. We demonstrated differences between young and older adults in task-dependent functional connectivity amongst neuromodulatory nuclei and afferent cortical regions that support attention and memory [8–12, 18, 19, 22]. Connectivity across these regions differed across ages, with a network dominated by target (go)-related activity in the young and distractor (no go)-related connectivity in older adults, with further pronounced changes in the functional roles of subcortical modulatory systems with each other and their afferent targets. As successful attentional orienting requires complimentary salience and distractor signals, we newly demonstrate that the push and pull between these signals switches with age with a marked difference in the functional role of subcortical neuromodulatory systems that contribute to both healthy and pathological neurocognitive aging [1, 13–17, 34–37]. This highlights the critical need to characterize the changes of neuromodulatory systems across the adult lifespan in health and disease and potential interventions to tune their contributions to the brain dynamics supporting cognition.

Our results show direct contributions of subcortical neuromodulatory nuclei and their cortical afferents on differential processing of target and distractor events, highlighting how functional coupling between these regions supports moment-to-moment attentional and behavioral salience. We restricted our analysis to nodes with known structural connectivity [8, 9, 11, 21, 51, 59–63] and found significant task-related functional coupling between them. As expected, these results indicate coupling of regions with synaptic connections [64], replicates previous findings in young adults of resting-state functional connectivity between many of these regions [12, 24, 39], and replicates a previous study using the same task which found LC-HPC functional connectivity during target trials [49]. As such, our results additionally inform hypotheses about the synergistic role that the LC and nbM perform together to alter large-scale brain states. As the NE and ACh systems generate flexible brain states, it follows that these two systems require functional coupling to allow for rapid fluctuations in attention [1, 31]. This is evidenced by large-scale network shifts from phasic LC and nbM firing covarying with the strength of the white-matter connectivity between the LC and nbM [65], consistent with our evidence of strong LC-nbM functional connectivity during rapid task switching.

These results also reveal prominent age differences in the interactions between neuromodulatory nuclei. Further, the direction of the LC-nbM task-dependent age effects depended on which region was chosen as the seed region. This could suggest that specific subregions of the nbM and LC may have differential task-dependent functional connectivity patterns, as there exists anatomical and functional heterogeneity in their selectivity for modulating cortical activity [66–68]. Because NE and ACh are posited to support cognitive flexibility in opposite ways [30], it follows that there are important task-related connectivity switches between these nuclei that may depend on the specific projection sites. Unlike previous studies which assessed age-related functional connectivity amongst this network during rest [24], we demonstrate functional coupling within this network that supports attentional switching modulated by noradrenergic and cholinergic coactivation with several structurally connected regions. Our work builds on prior work by showing that there are trial-to-trial fluctuations in functional connectivity between subcortical nuclei and their cortical afferents related to attention and behavioral salience, which dramatically change with age.

Despite the age-related alterations in this network, older adults in this study had no diagnosed cognitive deficits and similar task performance as younger adults. Although evidence indicates cognitive decline with aging, such as with response time and distraction detection, other domains are preserved in old age, such as attentional control and decreased mind-wandering [69–71]. These inconsistencies may be a result of large variability in the older population and educational experience and socioeconomic status might mediate this heterogeneity [71]. Though we cannot directly relate individual variability of our older sample to the age-related connectivity differences in these neuromodulatory networks, our results suggest that regions responsible for attentional and behavioral salience dynamically differ across the lifespan. Further work is needed to characterize the relationship between heterogeneous cognitive outcomes with changes in these regions.

Although aspects of attention may be preserved with old age [71–73], much evidence indicates age-related deficits [5–7]. We demonstrate a drastically altered network structure in older adults which may account for some of these age-related changes. For example, the altered connectivity and greater functional coupling during distractors relative to targets may result from prolonged processing of distractors, weakened distractor detection [6], and reduced inhibitory filtering of distractions [72, 74]. Our results also indicate that older adults have stronger LC-nbM coupling supporting salience signals but weaker nbM-HPC coupling supporting distractor signals compared to earlier in life, suggesting that this reversal in task-related functional connectivity reflects a reconfigured attentional network that may be the origin of stronger attentional gain without tuning, i.e., greater amplification of both targets and distractors [44] and thus reduced distractor filtering in old age [72]. The functional connections between the LC and nbM additionally become strengthened, consistent with the proposal that attentional switching and uncertainty are synergistically supported by NE and ACh systems [30, 31].

Despite being potentially compensatory in older adults, this altered network likely creates a vulnerable system [72, 73]. The LC and BF are some of the first regions to show evidence of structural and functional alterations with Alzheimer’s Disease [13–17, 34–36, 75, 76]. Our study builds on a body of literature showing significant changes in these neuromodulatory systems in healthy and pathological aging. Although our healthy older adult sample did not include any cognitive impairment or serious neurological disease, there were likely many cases of prodromal neurodegenerative disease since roughly 50% of individuals who live into their 90s will be diagnosed with Alzheimer’s Disease [77]. Investigating the functional coupling of this network in older adults diagnosed with mild cognitive impairment (MCI) and Alzheimer’s Disease will inform how disease onset and progression relates to restructuring of this network along the same timescale. Given that functional connectivity measured with fMRI is strongly correlated with the underlying structural connectome [55, 78], we would expect that major structural changes in MCI and Alzheimer's Disease would subsequently interfere with this attention-related functional network. Measuring this network in older adults with age-related cognitive impairment will identify differences between healthy and impaired elderly adults, which can better inform potential interventions targeting this subcortical brain network [38, 40, 41, 78–80].

Our study did contain a response inhibition component in addition to attentional processing. Several studies found that motor response inhibition is mediated by subcortical regions and large-scale functional networks [81–85], which were moderated by age [86]. These studies used a stop signal task (SST) in which most trials are target trials, setting up a prepotent response tendency with occasional stop trials requiring response inhibition. Although the SST employs similar trial conditions, in our task, with equiprobable trials, successful completion requires active attentional monitoring of each trial, as there is no learned actional tendency across trials. Inhibition-related activity is reduced in equiprobable go/no-go tasks [87], and the task we used has been shown to elicit increased pupillary responses even without overt behavior [44], indicating that the neural mechanisms controlling inhibitory control cannot account for our results.

Having identified significant changes in functional coupling across several subcortical neuromodulatory nuclei across young and old adults, our results provide a putative target for non-invasive stimulation and other rehabilitative techniques that have demonstrated clinical efficacy via modulating functional networks. For example, vagal nerve stimulation (VNS), a non-invasive stimulation procedure targeting the vagus nerve, is thought to involve the locus coeruleus-norepinephrine system [80]. VNS has been applied across a range of neuropsychiatric disorders and its efficacy in refractory epilepsy, for example, is in part due to altering functional networks [88]. Within the context of the attention-dependent changes in the subcortical network we observed across young and old adults, VNS could be used to modulate cortical networks by engaging the locus coeruleus [80]. One of the important needs is to evaluate and intervene on the health of the LC in middle age, before its downward trajectory in older age [33–36, 75]. Assessing LC health and its potential plasticity in midlife could be achieved by leveraging the assessment of the subcortical modulatory network interactions we have shown here, in combination with VNS.

Several limitations to the current work can be addressed in future work. First, our dataset is undersampled within the 46–65 years old middle age group and has high variance. As many structural and functional deficits can already be seen in middle age, investigating this age group is of great importance for interventional and preventative measures against age-related cognitive impairment [19]. Our preliminary analyses including middle-aged adults indicate potential nonlinear changes in subcortical task-dependent functional coupling representing an important time point in lifespan development (Supplemental Figs 1-3). Future work with greater sampling across the middle-aged range is a vital next step to assessing changes to this functional network in midlife. With more equal sampling across age groups, it would be possible to look at these functional differences across the continuum of age rather than in discrete age groupings.

An additional limitation is that we focused on task-related networks and do not have resting-state data. Resting-state functional connectivity has been shown to change significantly with age [89, 90]; thus, with resting-state and task fMRI we would be able to determine conventional functional connectivity metrics between these ROIs, which would give us information about baseline connectivity between these regions. Baseline functional connectivity of networks at rest has been additionally shown to shape functional networks during a task [91, 92]; therefore, comparing resting-state and task-based functional connectivity between these subcortical nuclei and across the lifespan is an important future research question. Further, although gPPI affords us the ability to detect task-dependent differences in functional connectivity in certain trial conditions relative to others, we cannot determine the absolute functional connectivity relationship that the task-dependent functional connectivity switches from when engaged in this orienting task.

In conclusion, we find that the major sources of NE and ACh in the brain and their known afferent target structures coordinate to support changing attentional and behavioral salience and do so differently across the lifespan. In old age, this apparently stable network shifts from behaviorally relevant events in young adults, with a notable strengthening of the mutual connectivity between neuromodulatory nuclei, to behaviorally irrelevant events in older adults. These findings provide new insights into how neuromodulatory nuclei support attention-state dependent functional coupling, and how they reorganize during healthy aging, serving as potential targets for assessments and interventions to maintain healthy cognition across the adult lifespan.

## Supplementary Information

Below is the link to the electronic supplementary material.

Supplementary file1 (PDF 295 KB)

## Data Availability

The prospectively acquired research data are available from the corresponding author on reasonable request.
