# Supplementary material for: Attention-dependent coupling with forebrain and brainstem neuromodulatory nuclei differs across the lifespan
Source: GeroScience. 2025 Mar 4;47(3):4301–20. doi: 10.1007/s11357-025-01582-0 (PMC12181466; doi:10.1007/s11357-025-01582-0)
Supplement: Supplementary file 1 — Supplementary file1 (PDF 295 KB) [file 11357_2025_1582_MOESM1_ESM.pdf]

Supplementary Figures

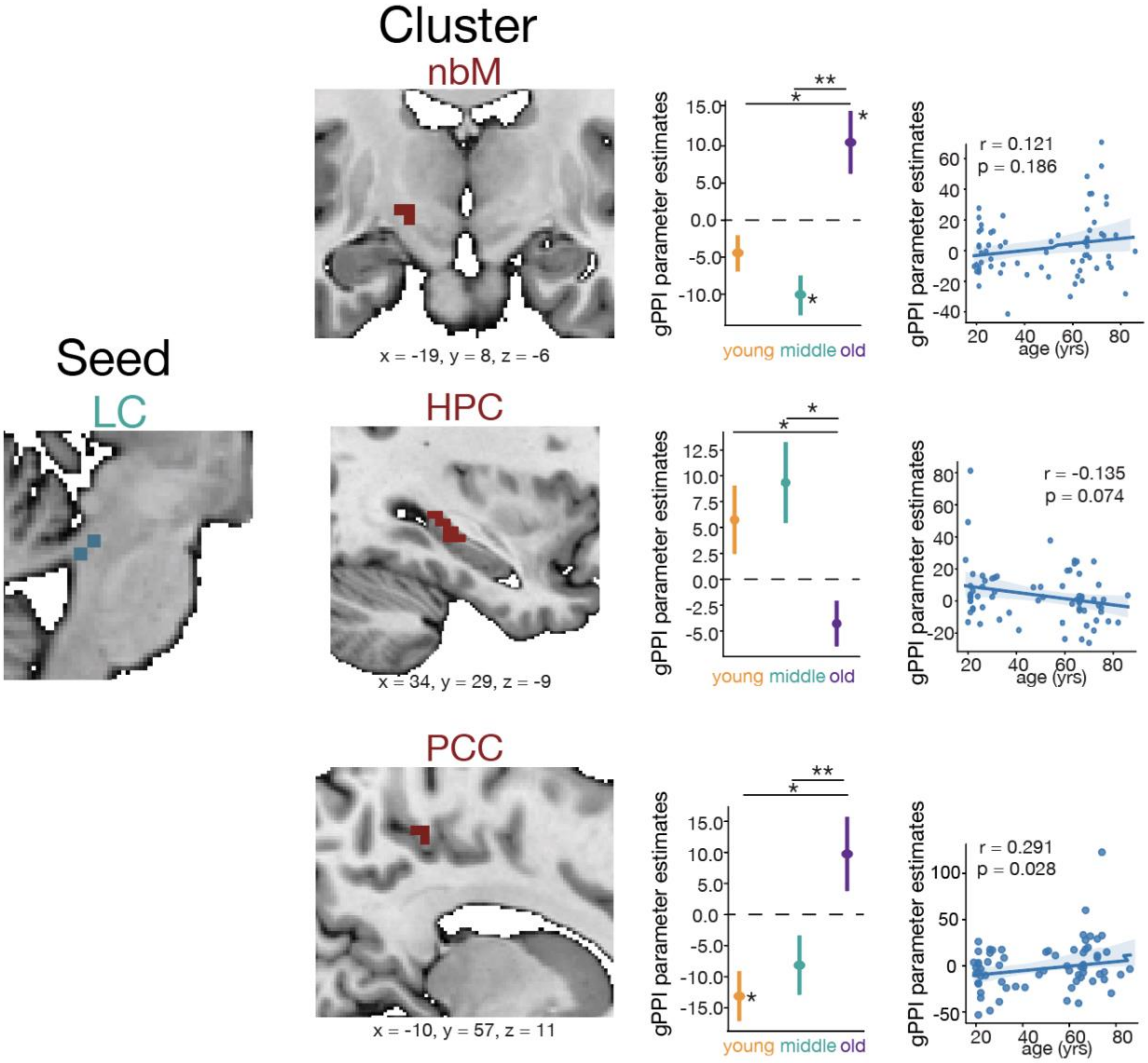

Supplemental Fig. 1 Exploratory LC-seed task-dependent functional connectivity with young, middle-aged, and older adults. Generalized psychophysiological interaction (gPPI) parameter estimates were extracted from the voxels within significant ROIs from a linear mixed effects model including the middle-aged adults (turquoise)

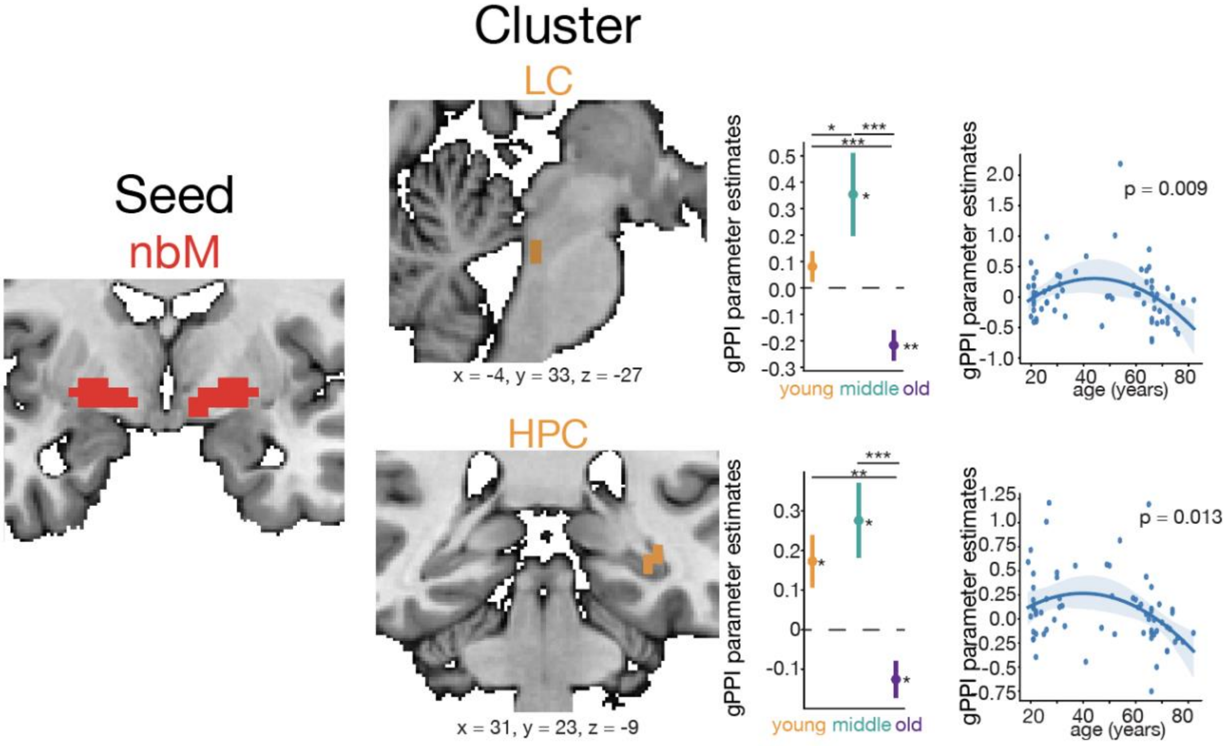

Supplemental Fig. 2 Exploratory nbM-seed task-dependent functional connectivity with young, middle-aged, and older adults. Generalized psychophysiological interaction (gPPI) parameter estimates were extracted from the voxels within significant ROIs from a linear mixed effects model including the middle-aged adults (turquoise)

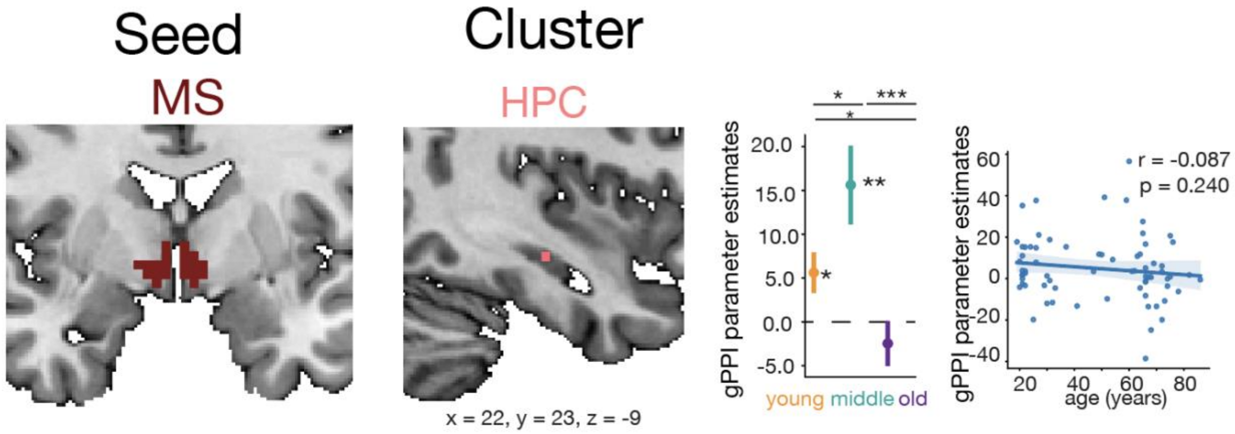

Supplemental Fig. 3 Exploratory MS-seed task-dependent functional connectivity with young, middle-aged, and older adults. Generalized psychophysiological interaction (gPPI) parameter estimates were extracted from the voxels within significant ROIs from a linear mixed effects model including the middle-aged adults (turquoise)
